# Metalens Enables Parallel Chromatic Confocal Imaging over a Millimeter-Scale Depth Range with an Axial Space–Bandwidth Product of 68

**DOI:** 10.1021/acs.nanolett.6c01339

**Published:** 2026-05-16

**Authors:** Yu-Jie Lin, Linghan Zhao, Md Tarek Rahman, Bofeng Liu, Guoyu Ding, Tunan Xia, Jinkai Yang, Xingjie Ni, Zhiwen Liu

**Affiliations:** Department of Electrical Engineering, 8082 The Pennsylvania State University, University Park, Pennsylvania 16802, United States

**Keywords:** metalens, chromatic confocal imaging, space−bandwidth product, surface profilometry, chromatic dispersion

## Abstract

Confocal microscopy provides optical sectioning and micrometer-scale resolution but remains constrained by mechanical axial scanning and bulky optics. Leveraging the intrinsic chromatic dispersion of a custom-designed metalens, we demonstrate parallel confocal depth imaging over a 1.3 mm axial range within a 200 nm visible bandwidth by encoding wavelengths into different axial positions to eliminate mechanical axial scanning, which yields an axial space–bandwidth product (ASBP) of ≈68. A single-mode fiber functions simultaneously as an illumination/collection probe and confocal pinhole, forming a compact design. The system achieves an axial resolution of 11.4 μm (axial sensitivity of 0.15 μm) and a lateral resolution of 2.19 μm near the optimal wavelength of 530 nm. The parallel confocal imaging capability across an extended depth is further validated by depth-resolved imaging and surface profile measurement. This compact, label-free platform underscores the potential of meta-optical chromatic confocal imaging for large-depth, high-resolution microscopic applications.

## Graphical abstract

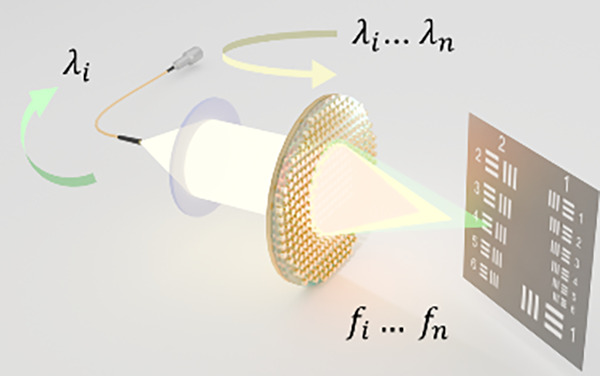

Confocal imaging is a powerful technique for non-destructive three-dimensional (3D) reconstruction.^1^ Employing a pinhole to reject out-of-focus light and collect only the in-focus component enables high-contrast imaging with optical sectioning capability^2^ and has been widely applied in fields ranging from industrial inspection to biomedical imaging.^3–7^ However, conventional confocal microscopy still relies on point-by-point axial scanning, thereby limiting imaging speed.^8–10^ For applications such as surface profilometry and endoscope-enabled surgery, rapid retrieval of axial information is critical.^11,12^ To address these constraints, various strategies have been developed. For example, electrically focus-tunable lenses can achieve scanning rates up to 100 Hz, while acoustic gradient-index lenses have demonstrated performance in the hundreds of kilohertz range.^13–15^

Despite these advances, such approaches still require active axial modulation. Chromatic confocal imaging offers a different modality that eliminates the need for axial scanning. This technique extends wavelength division multiplexing to imaging by exploiting the chromatic dispersion of optical materials, whereby different wavelength components of broadband illumination are focused on distinct axial positions, forming a continuous spectral–spatial encoding relationship across the operating bandwidth. By analysis of the reflected spectrum from the sample, the axial information can be directly retrieved without physical axial scanning. The concept was first demonstrated by Browne et al. in 1992 through chromatic confocal profilometry.^16^ Subsequent developments extended this approach by utilizing chromatic dispersion in various optical configurations, including singlet lenses, multi-element aspheric systems, and Fresnel zone plates.^17–22^ Although these works successfully eliminated axial scanning, they continued to rely on bulky optical components, imposing inherent limitations on the system size, alignment tolerance, and integration, particularly for space-constrained applications, such as confocal endoscopy.^23,24^

Meta-optics offers a compelling solution for miniaturizing imaging systems. Composed of ultrathin, subwavelength-structured elements, this technology can precisely manipulate the optical phase while replacing conventional bulky elements, such as lenses.^25–27^ Applications have been reported in diverse areas, such as biomedical imaging, hyperspectral analysis, holography, 3D reconstruction, and functional optical devices.^28–35^ Recently, researchers implemented a dispersive metalens in a chromatic confocal imaging system, demonstrating its compact form factor and depth-encoding capability.^31,36^ However, currently the number of optically resolvable axial sampling points, also known as the axial space–bandwidth product (ASBP), was restricted to values around 10, constraining the system’s ability for dense volumetric reconstruction. Limited axial information throughput may hinder a variety of applications, such as epithelial profiling in confocal endomicroscopy, semiconductor metrology, and 3D inspection of micro-optical components.^23,37,38^

In this work, we present a metalens-based chromatic confocal imaging system that achieves an ASBP of approximately 68 over a depth range of 1.3 mm, representing a substantial improvement over previously reported chromatic confocal systems. Over a 200 nm visible bandwidth, the system further demonstrates an axial sensitivity of 0.15 μm and a lateral resolution of 2.19 μm. A single-mode fiber (SMF) simultaneously serves as both the illumination probe and the confocal pinhole for signal collection, simplifying the optical configuration while preserving effective sectioning performance. These results highlight the potential of integrating meta-optics with chromatic confocal imaging to achieve large-axial-range, high-resolution 3D reconstruction within a compact architecture.

An overview of the working principle is shown in Figure 1. This schematic highlights how chromatic dispersion is harnessed to enable wavelength division multiplexing in confocal imaging or to encode depth information into a spectrum. A broadband supercontinuum source is delivered through a single-mode fiber to illuminate the metalens, which introduces chromatic dispersion and focuses different spectral components at distinct axial positions across an extended focal range (along the optical axis *z*), thereby creating a continuous series of focal planes, forming a “rainbow” of color-coded layers along the depth direction. When a sample is placed within this depth-encoded region, at each wavelength, light reflected from the focal plane is efficiently recoupled back into the same single-mode fiber, while light from out-of-focus regions is strongly suppressed and spatially filtered by the fiber (core diameter = 3.0 μm), which acts as a built-in confocal pinhole. By analysis of the reflected spectrum, the system translates each depth position into a corresponding peak wavelength, effectively using the wavelength as a “ruler” for depth. This inherent spatial–spectral mapping, arising from the combination of metalens chromatic dispersion and fiber-based confocal detection, enables access to the object’s depth information through only lateral scans. In other words, 3D structural information, such as surface profilometry and optical sectioning, can be achieved without axial scanning.

**Figure 1:**
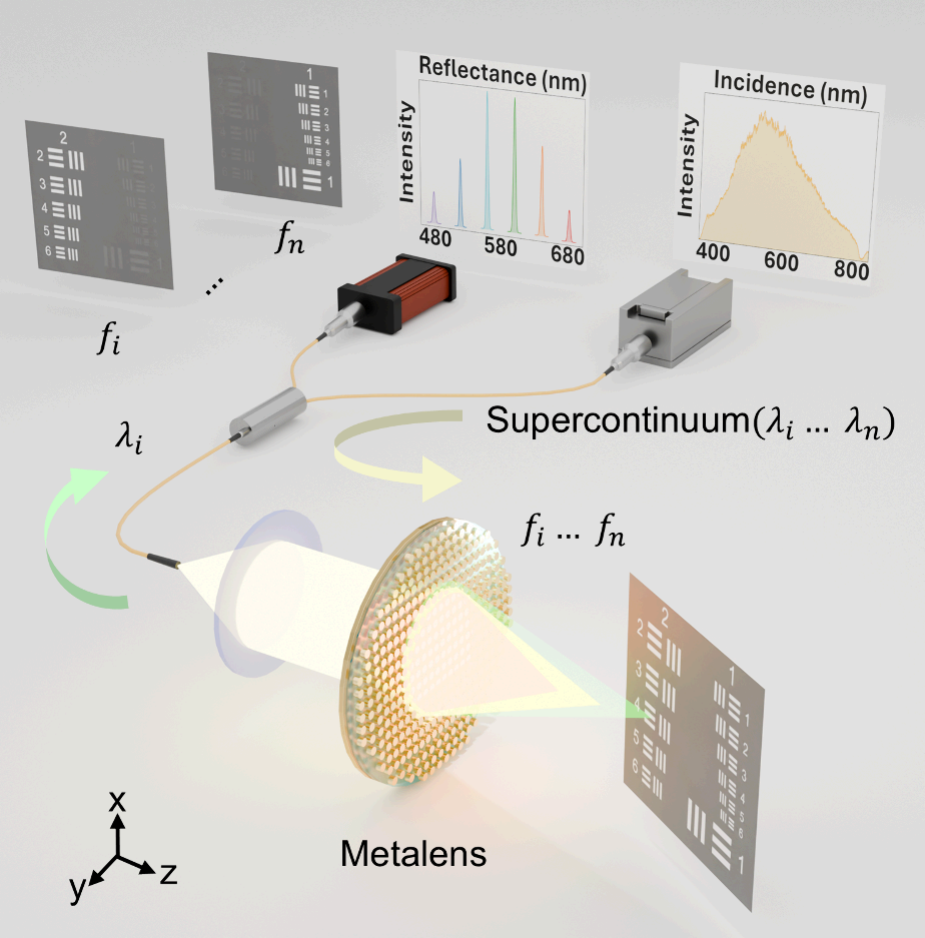
Schematic illustrating the working principle of the metalens-based confocal imaging system. A broadband supercontinuum source is delivered through a fiber to illuminate the metalens, which focuses different wavelengths at distinct axial positions to create a depth-encoding region. In-focus reflected light is recoupled into the fiber, an intrinsic confocal pinhole, and analyzed by a spectrometer, enabling optical sectioning and parallel axial imaging without mechanical scanning.

We designed a metalens with a central design wavelength (λ_0_) of 530 nm and focal length (f_0_) of 5 mm. Its ideal phase profile (at the wavelength λ) along the radial coordinate is given by

1 $$\phi \left(r\right)=\frac{2\pi }{\lambda }\left(\sqrt{r^{2}+f^{2}}-f\right)$$

where *f* denotes the wavelength-dependent focal length and follows:

2 $$f\left(\lambda \right)=\frac{f_{0}\lambda_{0}}{\lambda }$$

which establishes a mapping between the wavelength and axial focal position, enabling depth encoding in the subsequent chromatic confocal imaging implementation.

In high-NA regions of the metalens, where the phase gradient becomes steep, fixed-period sampling of meta-atoms leads to phase undersampling and degrades focusing performance. To mitigate this effect, we employed a Fresnel-zone wrapping approach, mapping the continuous phase distribution into the range (0, 2π). This strategy maintains an approximately linear phase variation even in high-gradient regions, effectively forming a local grating that redirects light toward the desired angle.^39^ Within each Fresnel zone, the geometry and placement of the meta-atoms were optimized to maximize diffraction efficiency into the target propagation direction, thereby realizing a high-efficiency linear phase ramp.

Silicon nitride was selected as the meta-atom material because of its high refractive index and low optical absorption across the visible spectrum (see Supporting Information S1). The fabricated metalens (Figure 3c) consists of 900 nm tall SiN circular nanopillars with a lattice period of 200 nm patterned on a silica substrate via electron-beam lithography. The symmetric nanopillar geometry ensures polarization-insensitive performance (see Supporting Information S2). The phase and transmittance response of the meta-atoms were numerically modeled using finite-element simulations in COMSOL Multiphysics (see Supporting Information S3).

To quantitatively establish the wavelength–depth relationship for the chromatic confocal system, a longitudinal mirror scan was performed along the *z* axis. The measured spectral response is shown in Figure 2a, where each wavelength component reaches its focus at a different axial position, forming a bright diagonal line that clearly demonstrates the system’s depth encoding. Across the full measurement range, the system covers approximately 1.3 mm in depth over a 200 nm visible bandwidth. By extracting the peak wavelength at each axial position and plotting its inverse against depth, we obtain a highly linear relationship (Figure 2b), which can be well-fitted by a linear model (*R* ^2^ = 0.997).

3 $$z\left(\lambda \right)=a\frac{1}{\lambda }+b$$

This dependence forms the basis of the depth encoding for subsequent 3D reconstruction. Such linearity is not incidental; it directly reflects the chromatic dispersion of the metalens, which translates broadband illumination into a continuous focal shift along the optical axis (*z*).

**Figure 2:**
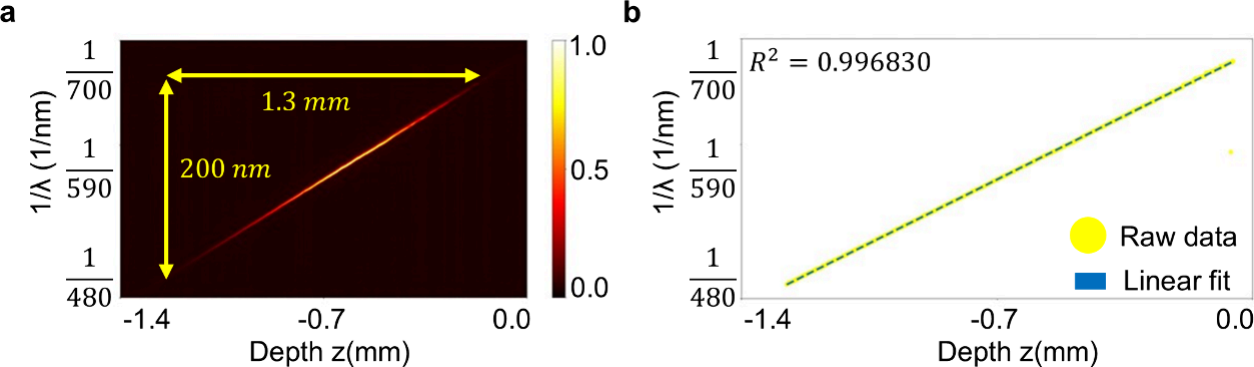
(a) Calibration with a longitudinal mirror scan reveals an axial depth range of 1.3 mm within a 200 nm visible spectral window. (b) Reflected peak wavelength is inversely proportional to the axial position, which can be fitted by a linear model $z\left(\lambda \right)=a\frac{1}{\lambda }+b$ , where *a* = −2.176872 × 10^3^ (mm nm) and *b* = 3.181622 (mm). The fitted model yields a coefficient of determination *R* ^2^ = 0.997, demonstrating consistency with the dispersion relationship in eq 2.

The metalens focusing performance was characterized by illuminating with a collimated beam (beam diameter = 1.4 mm) and recording focal intensity distributions at tunable wavelengths from 530 to 680 nm (50 nm increments) (see Supporting Information S4). The axial PSFs (Figure 3a, left) show wavelength-dependent focal shifts spanning ∼1.0 mm over this spectral band, and the lateral PSFs (Figure 3a, right) remain diffraction-limited across the visible range. The lateral resolution, defined as the full width at half maximum (fwhm) of the one-dimensional PSF, reaches 1.98 μm at 530 nm (Figure 3b). Diffraction-limited focusing is preserved across all measured wavelengths (see Supporting Information S5), indicating that axial chromatic dispersion does not degrade lateral performance. The reduced axial span compared to the 1.3 mm imaging range arises from the narrower 530–680 nm bandwidth used in the PSF measurement. Moreover, we measured the focusing efficiency of the metalens at different wavelengths, which reaches 85.4% at 530 nm and has an average efficiency of about 56% across several representative wavelengths (see Supporting Information S3).

**Figure 3:**
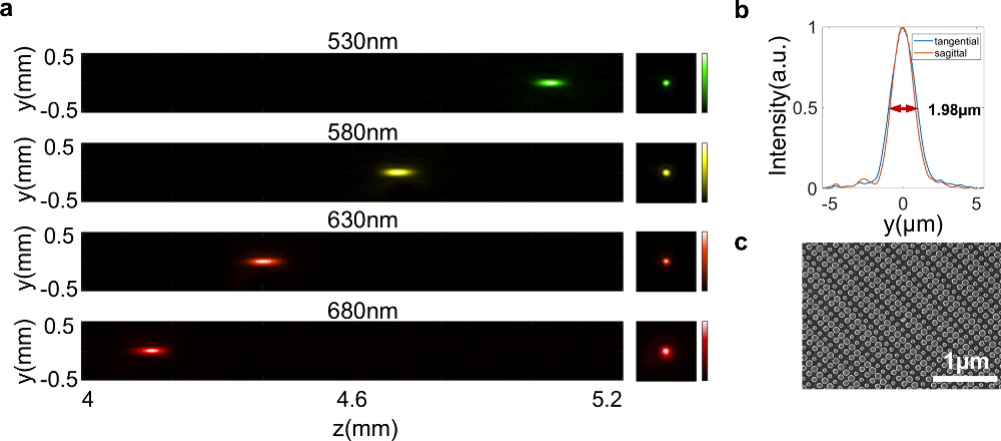
(a) Point spread function (PSF) measurements of the designed metalens in the axial (*z*) and lateral (*x* and *y*) directions, validating its diffraction-limited focusing performance. (b) Lateral full width at half maximum (fwhm) at the wavelength λ = 530 nm reaches 1.98 μm. (c) Scanning electron microscopy (SEM) image of the fabricated metalens.

Figure 4a shows the experimental setup of the chromatic confocal imaging system, incorporating the metalens. A broadband supercontinuum source is coupled into a single-mode fiber and delivered to its distal end. The emerging beam is collimated using a microscope objective (Newport M-10, *f* = 16.5 mm) and directed onto the metalens, which introduces chromatic dispersion to focus different wavelength components at distinct axial positions. When an object is placed within this depth-encoding region, the reflected light is recoupled into the same single-mode fiber, which simultaneously serves as a pinhole. The reflection spectrum was then measured using a spectrometer.

**Figure 4:**
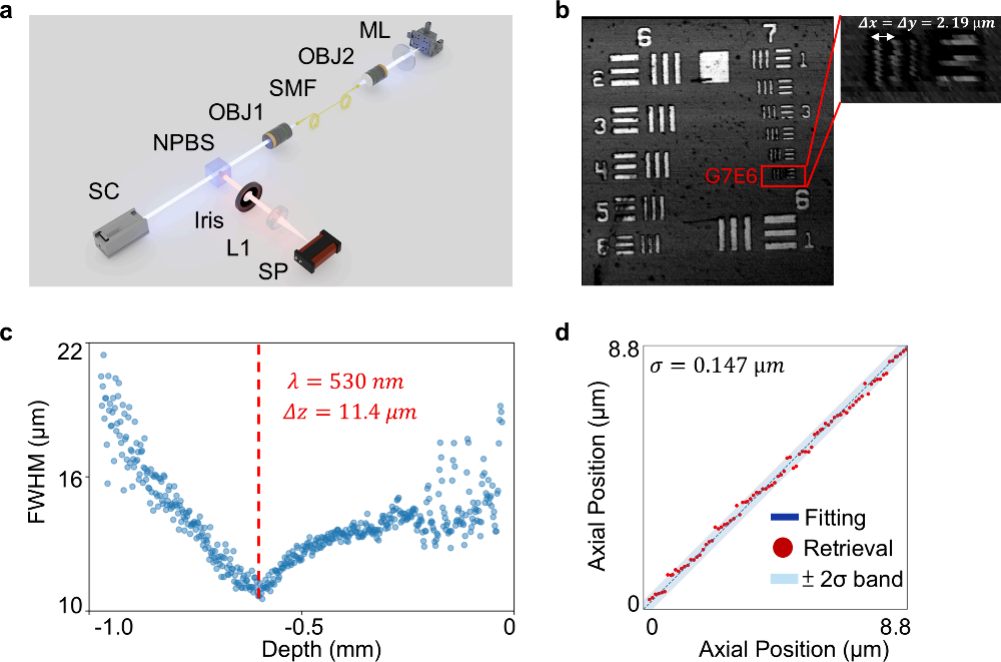
(a) Experimental setup of the chromatic confocal imaging system (SC, supercontinuum; SP, spectrometer; ML, metalens; component specifications in Supporting Information S6). (b) Lateral resolution test by scanning a USAF 1951 positive target, resolving the smallest feature (group 7, element 6) of 2.19 μm. (c) Axial resolution ranges between 10 and 22.4 μm and has a value of 11.4 μm at the wavelength λ = 530 nm. (d) Axial sensitivity test with a mirror scanned in 100 nm steps. Reconstructed depths (red dots) follow the motion-controller readouts (blue dashed, *y* = *x*) with σ = 0.15 μm (±0.30 μm at 2σ, 95% confidence); the light-blue band indicates the corresponding confidence range.

The system’s diffraction-limited imaging ability was validated by laterally scanning a USAF 1951 positive target with a step size of 0.8 μm. From the optical sectioning image acquired at the focal plane corresponding to the wavelength of λ = 576.59 nm (Figure 4b), the system resolved all features of groups 6 and 7, including the smallest element (group 7, element 6) with a feature period of 2.19 μm. It is worth mentioning that the measured fwhm of the PSF of the metalens (∼2 μm) at several representative wavelengths was also smaller than this feature size, consistent with the diffraction-limited imaging ability.

Axial resolution, defined as the fwhm of the reflected spectral peak at each axial position (see Supporting Information S7), calibrated using a longitudinal mirror scan, characterizes the system’s optical sectioning capability, specifically the minimal axial distance at which two closely spaced points can be distinguished. This metric directly determines the precision of depth discrimination and the quality of volumetric reconstruction. Across the central depth-encoding region (∼1.0 mm), the system maintains an axial resolution of 10.4–22.4 μm. At wavelengths near 530 nm, the axial resolution reaches a value of ≈11.4 μm (Figure 4c), in close agreement with the theoretically calculated value of ≈11.0 μm (see Supporting Information S8).

Integrating the local axial resolving capability over the 1.3 mm axial encoding range yields an experimentally determined axial space–bandwidth product ASBP_exp_ ≈ 68 (see Supporting Information S9). For comparison, a simple estimate can also be obtained using the representative axial resolution within the central depth-encoding region. Using the median axial resolution across this region Δ*z*_med_ = 19.4 μm, we obtain

$${\mathrm{ASBP}}_{\mathrm{est}}=\frac{\Delta f}{\Delta z_{\mathrm{med}}}\approx \frac{1.3\mathrm{mm}}{19.4\mathrm{\mu m}}\approx 67$$

These results confirm densely sampled axial encoding across the operating depth range without the need for active axial scanning.

Axial sensitivity quantifies the minimum axial displacement that can be detected through the depth–wavelength mapping. To measure this, a mirror was translated in 100 nm steps along the axial direction (*z*), and the axial sensitivity was determined from the standard deviation between the retrieved depths and their expected positions. At each axial location, the depth was retrieved by analyzing the reflected spectrum using a centroid-based wavelength extraction method (see Supporting Information S10), in which the centroid wavelength was converted to depth using the calibration function in eq 3. The expected value corresponds to the axial position reported by the motion controller. The retrieved depths (red dots) closely follow the controller readout (ideal line *y* = *x*, blue dashed) (Figure 4d), yielding a standard deviation of σ = 0.15 μm. The light-blue shaded region denotes the ±2σ (95%) confidence band around the ideal line, within which most reconstructed depths fall. The residual deviation primarily arises from open-loop mechanical actuation limits of the translational stage used for the characterization, representing a conservative estimate of the intrinsic optical sensitivity.

To demonstrate the system’s 3D reconstruction capability, a U.S. quarter was laterally scanned with a step size of 10 μm. The coin was intentionally tilted so that its left side was positioned closer to the metalens, corresponding to the focal plane of longer wavelength illumination. At each lateral position, a reflected spectrum was recorded, enabling two reconstruction modalities, surface profilometry and optical sectioning, to be derived from the same spectral data set using different processing strategies.

Figure 5a shows the raw peak reflectance intensity map. For each lateral position, the peak reflectance intensity was extracted from the measured spectrum. In this reflectance-type configuration, illumination incident on steep boundaries (e.g., engraved edges) redirects the reflected light away from the optical axis, causing it to fall outside the fiber’s acceptance cone and resulting in only weak background signals. Consequently, flat surfaces appear bright while engraved regions appear dim, naturally revealing the surface morphology of the coin.

**Figure 5:**
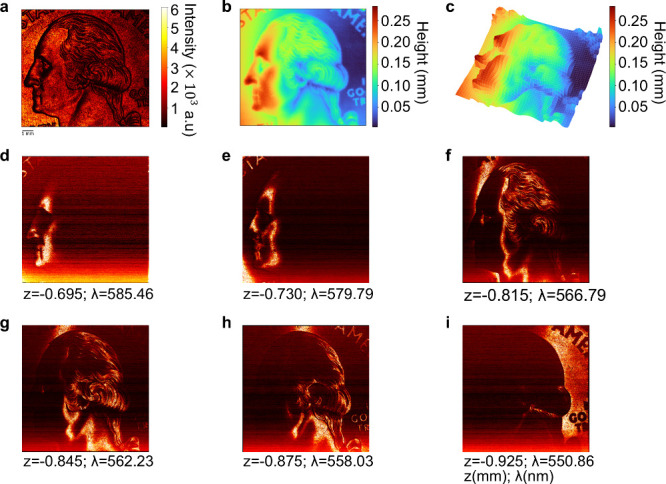
Surface profilometry and optical sectioning of a U.S. quarter (10 μm lateral step size). (a) Reflectance intensity map showing the overall surface morphology, where steep boundaries exhibit low reflectance. (b) Depth-resolved surface profile reconstructed using the calibrated *z*(λ) mapping. The profile is re-zeroed by setting the most negative axial position to 0 for clarity. A turbo colormap is used to enhance height contrast. (c) 3D contour representation of the reconstructed surface. (d–i) En face optical sections at different axial depths retrieved directly from the original *z*(λ) values (without rezeroing): (d) −0.695 mm, (e) −0.730 mm, (f) −0.815 mm, (g) −0.845 mm, (h) −0.875 mm, and (i) −0.925 mm. Because the coin was slightly tilted laterally, the focal plane traversed from left to right across the sample. At shallower depths, only the left region was in focus and coupled into the single-mode fiber, whereas deeper focal planes revealed distinct features toward the right side.

For surface profilometry, spectra recorded at steep boundaries exhibit low reflectance and do not provide reliable peak information for axial localization. We therefore applied baseline flattening and a noise-robust peak-classification procedure to retain only spectra with valid confocal peaks (see Supporting Information S11). For each peak-detectable lateral position, the extracted peak wavelength was converted to depth using the calibrated wavelength–depth mapping function. The resulting height map, shown in Figure 5b, was subsequently re-zeroed by applying a constant axial offset such that the most negative retrieved axial position, corresponding to the point farthest from the metalens within the scanned region, was defined as 0 mm. This procedure preserves all relative height variations across the surface. The corresponding 3D surface reconstruction is presented as a contour plot in Figure 5c, reflecting the overall geometry of the coin.

Beyond surface profilometry, the system enables depth-resolved optical sectioning without mechanical axial scanning. When different sectioning wavelengths are selected and corresponding lateral intensity distributions are plotted, en face images at successive axial planes are obtained, as shown in Figure 5d–i. Due to the chromatic dispersion of the metalens, longer wavelengths are focused closer to the metalens, such that only features located near this shallow focal plane appear bright (left region of the coin), while out-of-focus regions are efficiently suppressed by the single-mode fiber acting as a confocal pinhole (Figure 5d). As the sectioning wavelength is progressively reduced, the focal plane shifts farther away from the metalens, causing the in-focus region to move laterally from left to right across the coin surface (Figure 5d–i). This sequential shift reveals distinct engraved features at different depths, spanning an axial range of approximately 0.23 mm within the scanned region. These results demonstrate effective confocal gating and densely sampled optical sectioning over a large depth range, achieved entirely without mechanical movement along the axial direction.

In chromatic confocal imaging, the ASBP quantifies the number of resolvable axial sampling points and is defined

4 $$\mathrm{ASBP}=\frac{\Delta f}{\delta z}$$

where Δ*f* is the chromatic focal shift across the operating bandwidth and the diffraction-limited axial resolution δ*z* is given by

5 $$\delta z=k\frac{\lambda }{n{\mathrm{NA}}^{2}}$$

where *n* is the refractive index of the surrounding medium (air, *n* = 1), NA is the numerical aperture of the metalens, and *k* is a configuration-dependent constant on the order of 1. For this discussion, we take λ = 530 nm for simplicity. Physically, ASBP quantifies the number of resolvable axial sections in chromatic confocal imaging or the axial information capacity of the system.

The achievable ASBP depends on both the numerical aperture of the metalens and the spectral resolution of the detector (in our work, the spectrometer).^40^ A finite spectral resolution (δλ) of the spectrometer defines an effective coherence length *L*_c_ = λ^2^/δλ, which bounds the optical path difference across the metalens aperture and thereby determines the maximum usable aperture radius and, hence, the effective numerical aperture. This condition can be expressed as (see Supporting Information S12 for derivation)

6 $$r\leq \frac{\lambda^{2}}{\delta \lambda \left(\frac{1-\sqrt{1-{\mathrm{NA}}^{2}}}{\mathrm{NA}}\right)}$$

where *r* is the radius of the aperture. This relation indicates that the spectrometer resolution determines the maximum useful metalens aperture radius permitted by the coherence constraint. Under our present resolution (δλ ≈ 0.39 nm), the coherence-limited aperture radius is ≈2.78 mm. In our implemented system, a 2.7 mm diameter collimated beam was used, yielding an effective NA of 0.26 and the experimental ASBP_exp_ ≈ 68.

The coherence constraint can also be expressed in terms of the spectrometer resolving power *R* ≡ λ/δλ, yielding

7 $$R\geq \frac{r\left(1-\sqrt{1-{\mathrm{NA}}^{2}}\right)}{\mathrm{NA}\cdot \lambda }$$

The metalens enables access to a high-NA regime, which is essential for maximizing axial information throughput.^41,42^ In the present implementation, we employ a portable spectrometer with a relatively coarse resolution. Accordingly, the aperture is limited within a coherence-compatible regime defined by the detection system. To fully utilize the available metalens numerical aperture (NA ≈ 0.93), a resolving power *R* of ≥1.8 × 10^4^ (corresponding to δλ ≤ 0.027 nm) is required. Increasing the spectral resolving power enables the full NA to be utilized, under which the system can potentially achieve ASBP > 1000, highlighting the scalability of high-NA metalenses for enhanced axial information capacity.

In summary, we demonstrate a metalens-based chromatic confocal imaging system that eliminates mechanical axial scanning by encoding depth directly into wavelength through chromatic dispersion. Near the optimal wavelength (530 nm), the system achieves an 11.4 μm axial resolution, ≈0.15 μm axial sensitivity, and 2.19 μm lateral resolution. A 1.3 mm imaging range is demonstrated with an axial space–bandwidth product of ≈68. A single-mode fiber serves as the illumination and collection probe while acting as the intrinsic confocal pinhole, enabling a compact and alignment-robust optical architecture.

Beyond the current implementation, the combination of millimeter-scale depth range, high ASBP, and a compact form factor underscores the practical potential of chromatic metalenses for miniaturized confocal imaging systems. Further improvements in optical throughput, particularly in scattering media and at steep interfaces, will extend the applicability to more demanding real-world applications.

## Supplementary Material



## Notes

Supporting Information S1–S12, including additional theoretical derivations, material characterization data, fabrication details, experimental procedures, and extended analysis (PDF)
