# Supplementary material for: Metalens Enables Parallel Chromatic Confocal Imaging over a Millimeter-Scale Depth Range with an Axial Space–Bandwidth Product of 68
Source: Nano Lett. 2026 May 16;26(21):7016–22. doi: 10.1021/acs.nanolett.6c01339 (PMC13237768; doi:10.1021/acs.nanolett.6c01339)
Supplement: Supplementary file 1 [file nl6c01339_si_001.pdf]

Supplementary Information

Metalens Enables Parallel Chromatic Confocal Imaging over mm-scale Depth Range with an Axial Space-Bandwidth Product of 68

Yu-Jie Lin[‡]*, Linghan Zhao*[‡]*, Md Tarek Rahman, Bofeng Liu, Guoyu Ding, Tunan Xia, Jinkai Yang, Xingjie Ni, and Zhiwen Liu*

Department of Electrical Engineering, The Pennsylvania State University, University Park,

Pennsylvania 16802, USA

1. Refractive index n and absorption coefficient k of silicon nitride.

The chosen material possesses low loss and high refractive index for fabricating high-efficiency chromatic metalens within the operating visible window (480-680 nm).

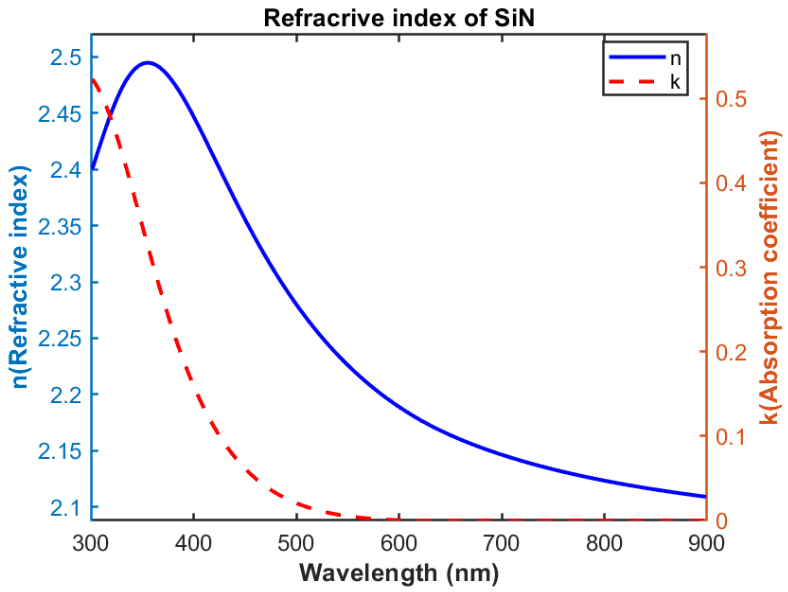

Figure S1. Measured refractive index and absorption coefficient of silicon nitride within the operation band.

2. Fabrication flow of the metalens, using conventional electron-beam lithography.

The fabrication process begins with substrate preparation to ensure a clean and smooth surface. Transparent fused silica substrates were selected due to their low optical absorption in the visible spectrum. The substrates were sequentially cleaned in acetone to remove organic contaminants, rinsed in isopropanol (IPA) to eliminate acetone residues, and immersed in Nanostrip 2× solution for 30 min to remove residual metallic contaminants.

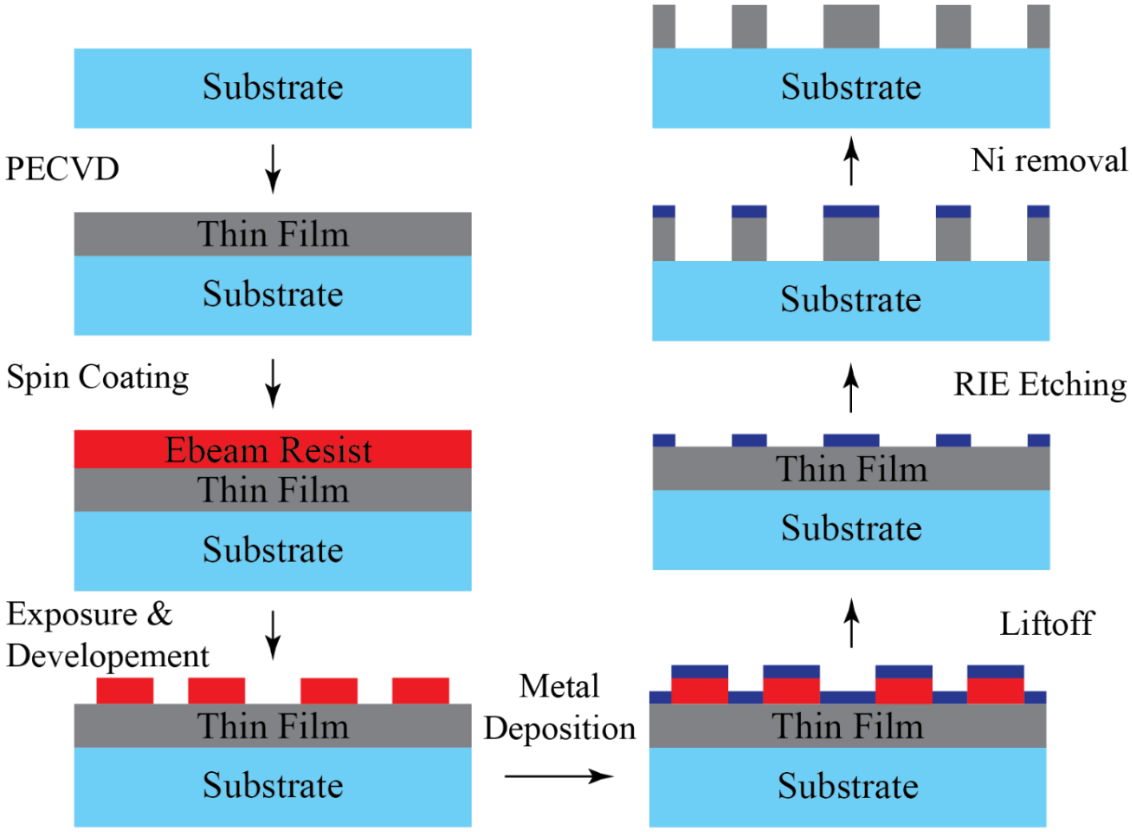

Figure S2. Fabrication flow of the metalens

A silicon nitride (SiN) thin film was deposited on the cleaned substrates using plasma-enhanced chemical vapor deposition (PECVD). The deposition parameters are summarized in Table S1.

Table S1. PECVD Deposition conditions for silicon nitride

Material	Gases	flow rate (sccm)	Pressure	Temperature	Power	Deposition Rate
Silicon Nitride	NH_3	100	3.5 Torr	300°C	300W	8.5 nm/sec
	SiH_4	150				

Electron-beam lithography was used to pattern the metalens design using a positive-tone resist (ZEP-520, 1:1 dilution). To improve resist adhesion, the substrate was treated with SurPass 4000 solution, an aqueous-based priming agent, followed by baking at 100 °C for 1 min. After cooling, ZEP resist was spin-coated at 4000 rpm and baked at 185 °C for 3 min, yielding a resist thickness of 110–120 nm. To improve surface conductivity during exposure, a 25 nm aluminum layer was

deposited by thermal evaporation. Pattern exposure was performed using a Raith EBPG 5200 system.

After exposure, the aluminum layer was removed using Microposit MF CD-26 developer prior to resist development. The exposed resist was developed in amyl acetate for 3 min and rinsed in IPA to terminate development.

A 40 nm nickel (Ni) hard mask was deposited by electron-beam evaporation. Lift-off was performed in anisole for 30 min, followed by 3 min ultrasonication to remove excess Ni. Finally, the substrate was immersed into IPA solution to remove anisole residue.

Reactive ion etching (RIE) was used to transfer the pattern into the SiN layer using CHF_3 and CF_4 gases. The etching parameters are summarized in Table S2.

Table S2. Dry etching conditions for silicon nitride

Material	Gases	flow rate (sccm)	Pressure	Temperature	Chuck Power, ICP Power	Etch Rate
Silicon Nitride	CHF_3	10	2.5 mTorr	25°C	50W, 600W	160 nm/min
	CF_4	30				

3. Optical response for meta-atoms and experimental focusing efficiency

3.1 Simulated phase and transmittance of the meta-atoms

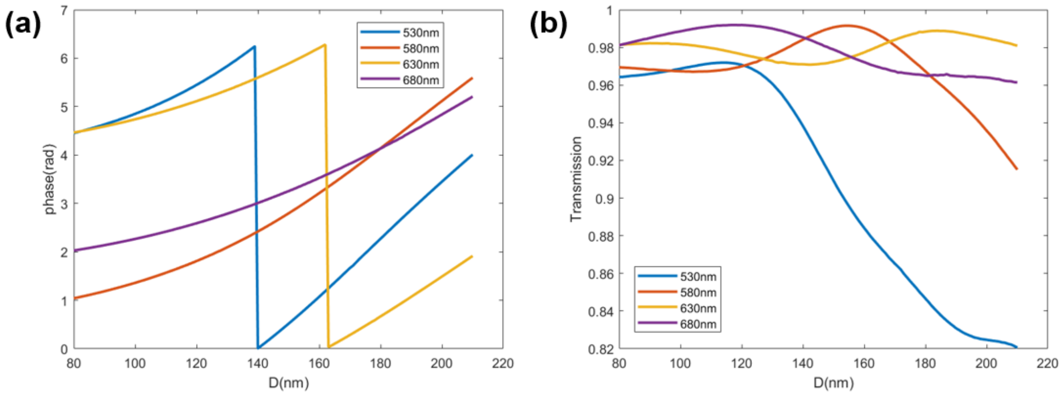

Figure S3. (a) phase and (b) transmission response of the designed meta-atom at 530nm, 580nm, 630nm and 680nm, respectively. x-axis: diameter of the meta-atom, y-axis: simulated phase or transmission response of the meta-atom.

3.2 Focusing efficiency measurement

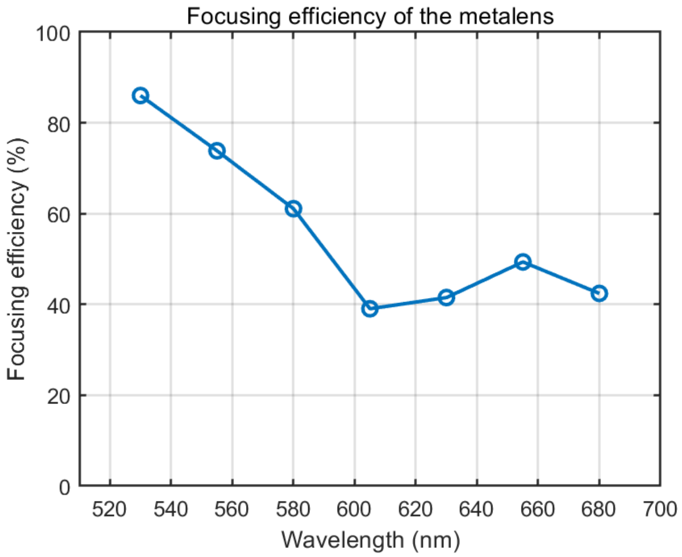

Figure S4. Measured focusing efficiency of the metalens at different wavelengths. The focusing efficiency was characterized at multiple wavelengths (530 nm, 555 nm, 580 nm, 605 nm, 630 nm, 655 nm, 680 nm). At each wavelength, the power at the focal spot was measured in repeated trials, and the efficiency was calculated from the averaged focal power normalized to the corresponding input power.

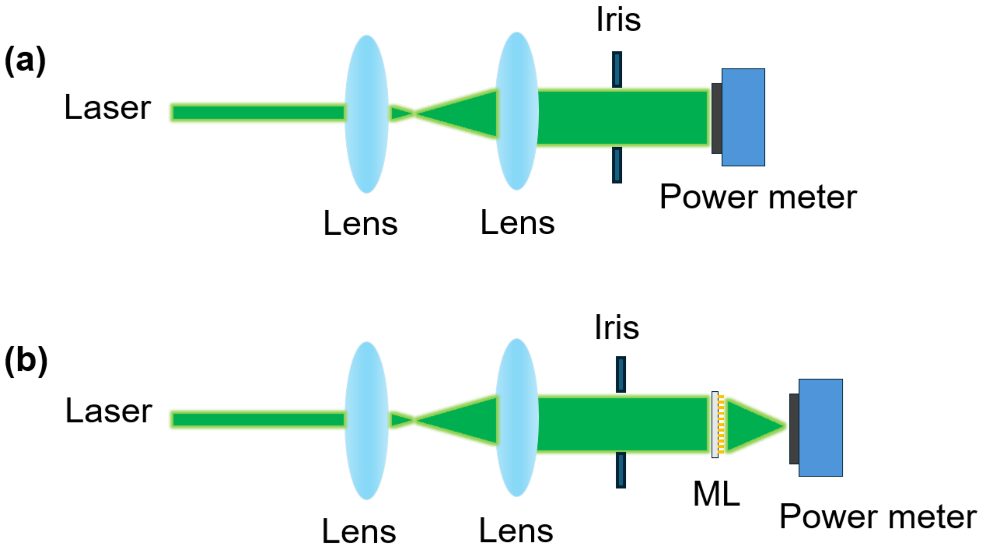

Figure S5. Schematic of the setup used for characterizing the focusing efficiency. (a) Reference measurement of the incident optical power through the iris aperture in the absence of the metalens. (b) Measurement of the optical power delivered by the metalens (ML) after insertion into the beam path. The focusing efficiency at each wavelength was calculated as the ratio of the

measured focal output power to the corresponding incident power. The focusing efficiency is defined as the optical power collected at the focus, normalized to the corresponding incident optical power on the clear aperture of the metalens.

4. Experimental setup for PSF test

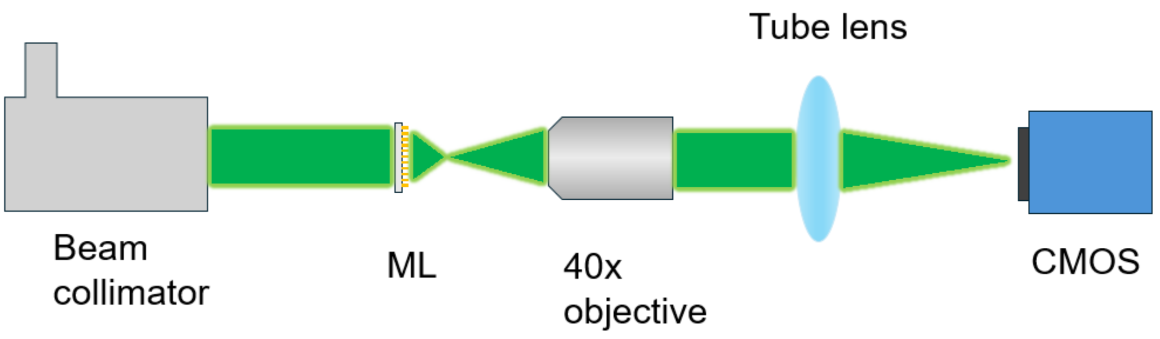

Figure S6. Schematic of metalens PSF measurements setup.

The metalens' PSF was measured using an infinity-corrected microscope imaging configuration. The illumination was provided by a femtosecond Ti:Sapphire laser (Coherent Chameleon Ultra II) together with an OPO/SHG system (Chameleon Compact OPO VIS, APE GmbH) to access different wavelengths. The output beam was expanded and collimated (RC12SMA-P01, Thorlabs; beam diameter ~ 13.2 mm) before illuminating the metalens. After transmission through the metalens, the focal distribution was collected by a high-NA objective (40×, NA = 0.95, ZEISS) and relayed by a 200-mm tube lens (TTL200-S8, Thorlabs) to a CMOS detector (DMK 23GP031, The Imaging Source; 400–1100 nm). The recorded intensity profiles at the camera plane were used to extract the PSF. For characterization, we performed z-scans to capture 200 slice layers between 4mm to 5.2mm, each consisting of 3 frames per layer.

5. PSF analysis for additional wavelengths

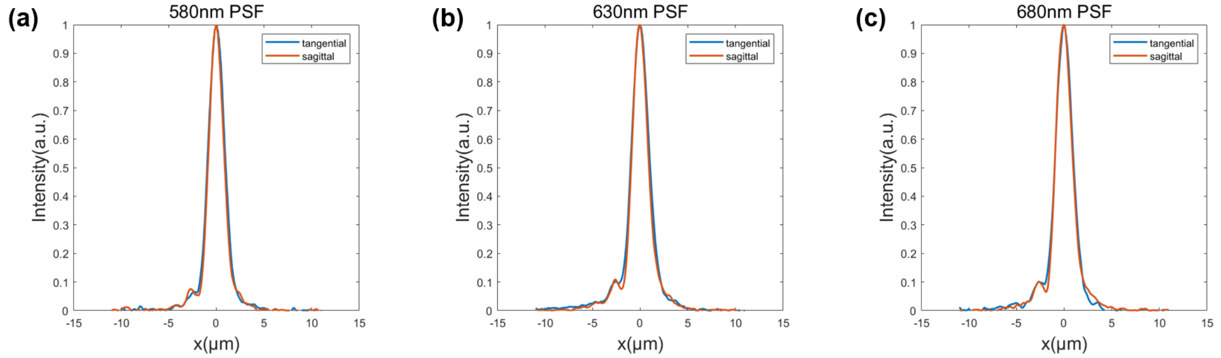

Figure S7. Normalized tangential and sagittal PSF profiles of the metalens at different wavelengths (Derived from Fig.3a) (a) 580 nm, (b) 630 nm, and (c) 680 nm.

Figure S7 shows the measured point spread funtion of metalens at 580nm, 630nm and 680nm, respectively. Their FWHMs along tangential and saggital direction are shown in Table S3:

Table S3. FWHM of PSF along tangential and sagittal direction at 580nm, 630nm and 680nm.

	Tangential FWHM	Sagittal FWHM
580nm	1.96 μm	1.79 μm
630nm	2.08 μm	1.88 μm
680nm	2.03 μm	1.95 μm

6. Components specification for the metalens-based chromatic confocal system

Broadband light from a supercontinuum source (SC; SuperK COMPACT, NKT Photonics; 450–2400 nm) is directed through a non-polarizing beamsplitter (NPBS; BS013, Thorlabs; 50:50, 400–700 nm) and coupled by a 40× objective (OBJ1; M40, Olympus; NA = 0.65) into a 2-m single-mode fiber (SMF; S405-XP, Thorlabs; 400–680 nm; Ø125 μm cladding, 2.5 μm core). At the distal end, the output light is collimated (OBJ 2; M-10x; Newport) before illuminating the metalens (ML), which introduces chromatic dispersion. Reflected light is recoupled into the SMF, where the fiber acts as an intrinsic confocal pinhole, then redirected by the NPBS and focused by a 100-mm lens (L1) into a spectrometer (SPEC; FLAME-S-VIS-NIR-ES, Ocean Optics) for depth-resolved spectral detection.

7. Definition of axial resolution

Each curve represents raw reflected spectrum (with wavelength converted to the calibrated depth position) at each axial position. The axial resolution is defined as the Full-Width at Half-Maximum (FWHM).

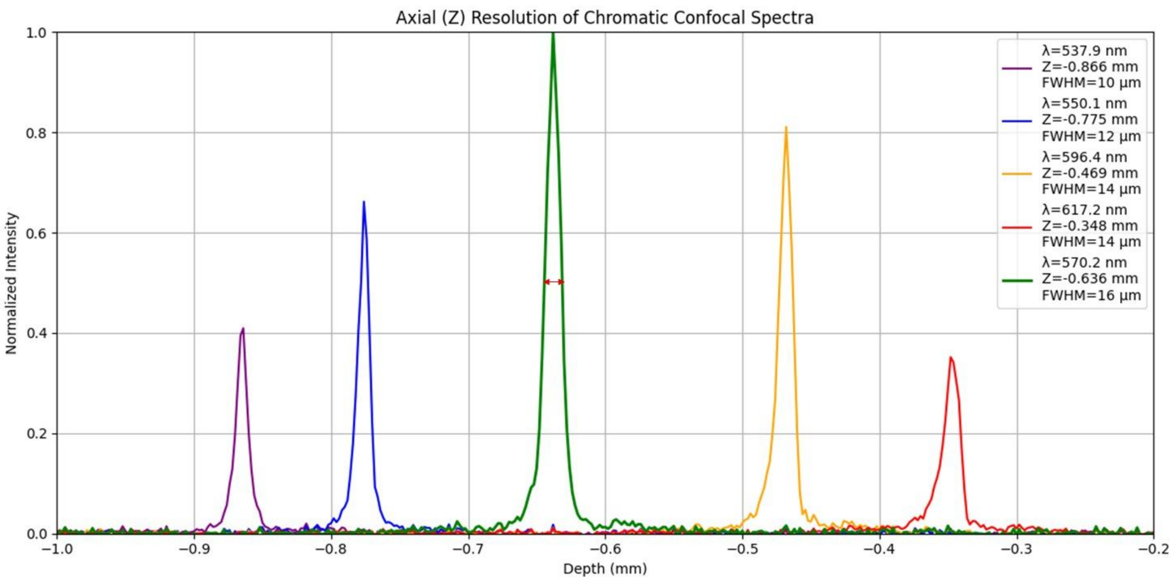

Figure S8. Definition of axial resolution by FWHM

8. Calculated axial resolution at the wavelength of 530 nm

$$\delta z = \frac{k\lambda}{NA^2} = \frac{1.4\lambda}{NA^2} = \frac{1.4 \times 0.53\ \mu m}{0.26^2} \approx 11.0\ \mu m \qquad (S1)$$

At the central design wavelength, the confocal configuration enhances the axial response by $\approx \sqrt{2}$ compared to the wide-field illumination. Therefore, we adopt $k = 1.4$ here. The numerical aperture is calculated under the collimated illumination (beam diameter ~ 2.7 mm) and the design focal length of the metalens (f_0 = 5 mm at λ_0 = 530 nm).

9. Experimental axial space–bandwidth product (ASBP)

The ASBP was evaluated from the measured spectral matrix $I(\lambda, z)$. Each axial position was fitted with a Gaussian to obtain the effective spectral width $\Delta\lambda_{\mathrm{eff}}$ after deconvolution of the spectrometer resolution. By integrating the ratio between the sampling interval and local spectral resolution across the analyzed band, the ASBP in the wavelength domain is expressed as:

$$ASBP_{exp} = \sum_i \frac{\Delta\lambda_{bin,i}}{\Delta\lambda_{eff,i}} \qquad (S2)$$

Here, $\Delta\lambda_{\mathrm{bin},i}$ denotes the spectral sampling interval between adjacent wavelength centers, estimated from neighboring peaks using the mid-point rule, and $\Delta\lambda_{\mathrm{eff},i}$ is the effective spectral resolution obtained from Gaussian-fitted linewidths after deconvolution of the spectrometer resolution.

10. Definition of centroid wavelength

The centroid wavelength λ_c at each axial position is calculated as:

$$\lambda_C = \frac{\sum_{\lambda_{min}}^{\lambda_{max}} \lambda \times I(\lambda)}{\sum_{\lambda_{min}}^{\lambda_{max}} I(\lambda)} \qquad (S3)$$

Here, λ denotes the discrete spectrometer sampling point corresponding to the reflected peak, and I(λ) is its normalized intensity, defined with respect to the maximum intensity of the peak. Because the low-intensity tails at both ends of the peak are unstable over time and are dominated by noise, only spectral points with I(λ)>0.2 are used for depth retrieval.

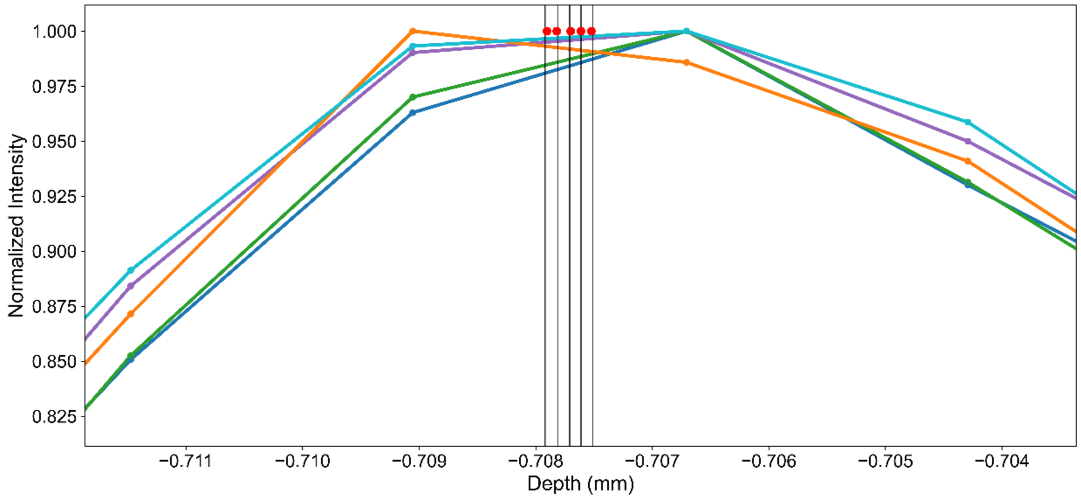

Figure S9. Axial positions retrieved from centroid wavelength and calibration function $z(\lambda)$, which align with the ground truth (black lines) well.

In Figure S9, representative red dots denote the reconstructed axial positions obtained from the calibration function $z(\lambda)$, where λ corresponds to the centroid wavelengths $\boldsymbol{\lambda_c}$ calculated in Eq. (S3). The black straight line indicates the ground-truth depth values reported by the motion controller, while the colored lines connecting the data points represent the raw spectra, converted into axial coordinates using the same calibration.

11. Surface-profile reconstruction from chromatic confocal spectrum

11.1 Overview:

To reconstruct the surface height profile of the quarter coin, each lateral scan position (x_i, y_i) provides one reflectance spectrum $S_i(\lambda)$. The axial position of the surface is encoded in the wavelength at which the spectrum reaches its in-focus maximum. However, raw spectrum exhibit baseline drifts, noise, and occasional missing peaks at steep boundaries. The following procedure converts raw spectra into a robust depth map $z(x, y)$.

11.2 Baseline Flattening

Raw reflectance spectra exhibit slow-varying background due to broadband illumination and stray reflections. This baseline is estimated using a Savitzky–Golay (SG) polynomial filter as shown below:

$$B(\lambda) = SG(S(\lambda)) \tag{S4}$$

$$S_{flat}(\lambda) = S(\lambda) - B(\lambda) \tag{S5}$$

We typically use:

- window length: 51–71 points
- polynomial order: 1–2

The flattened spectrum $S_{\text{flat}}(\lambda)$ retains the sharp confocal peak while removing the baseline.

11.3 Robust Peak Classification

Steep boundaries on the coin sometimes produce very weak or ambiguous peaks. To avoid false depth results, a classification rule is applied.

Noise estimation

Noise level is estimated from the median absolute deviation of the flattened spectrum:

$$\sigma_{noise} = 1.4826 \cdot median(|\, S_{flat}(\lambda) - median(S_{flat}) \,|) \tag{S6}$$

Peak detection rule

A wavelength index λ_p is accepted as a valid confocal peak if:

1. Peak height exceeds minimum SNR requirement

$$S_{flat}(\lambda_p) > Max(H_{abs}, \alpha\sigma_{noise}) \tag{S7}$$

2. Prominence exceeds threshold

$$Prominence(\lambda_p) > \beta\sigma_{noise} \tag{S8}$$

Here, Peak prominence is defined as the vertical distance between the peak maximum and the higher of the two adjacent local minima on either side of the peak. It quantifies how distinctly a confocal peak stands out from its local spectral background, rather than its absolute intensity.

Typical values:

- absolute height $H_{\text{abs}} = 800$, which is the intensity threshold applied to the baseline-flattened spectrum, serving as a lower bound to reject extremely weak signals regardless of the estimated noise level.

- SNR factor $\alpha = 5$,

- prominence factor $\beta = 1$

Points failing either criterion are labeled as no-peak and assigned NaN, indicating insufficient confocal return.

11.4 Wavelength-to-depth conversion

The metalens is calibrated by measuring the focal wavelength as a function of axial position. The depth z corresponding to a detected peak wavelength λ_p is obtained by inverting the cubic calibration polynomial:

$$\lambda(z) = az^3 + bz^2 + cz + d \qquad (S9)$$

with experimentally fitted coefficients:

$$(a, b, c, d) = (-2.12,\ 32.88,\ 207.70,\ 713.12).$$

We solve for z using a numerical root finder:

$$z = \lambda^{-1}(\lambda_p), z \in [z_{min}, z_{max}], \qquad (S10)$$

where a bracketing range $[-2\text{ mm}, 0\text{ mm}]$ ensures stable convergence.

11.5 Spatial reconstruction and interpolation

Each lateral point yields:

$$(x_i, y_i, z_i)$$

Because the acquisition grid is obtained using velocity-mode scanning, in which the translation stage moves continuously at a nominal constant speed during data acquisition rather than stopping at discrete step-and-settle positions, the recorded lateral sampling positions are not perfectly uniform due to motion jitter, acceleration transients, and timing uncertainty. To address this, the resulting point cloud is interpolated onto a uniform mesh:

$$z_{grid}(x, y) = griddata((x_i, y_i), z_i) \qquad (S11)$$

using linear interpolation with nearest-neighbor fill for missing points.

11.6 Final surface profile visualization

The reconstructed depth map is displayed using a perceptually uniform colormap (e.g., *hot*). The coin tilt appears as a global sloped background, while local morphology—hair strands, nose ridge, text engravings—is preserved with high axial sensitivity.

12. Derivation of the relationship between spectral resolution, lens aperture, and numerical aperture

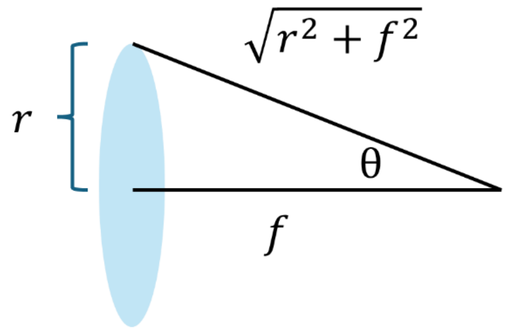

Figure S10. Geometry used to derive the coherence-limited aperture constraint. The marginal ray from the metalens edge (radius r) travels a distance $\sqrt{r^2 + f^2}$to the focus, forming an angle θ with the optical axis, while the on-axis ray travels a distance f. Their optical path difference ΔL determines the coherence-limited maximum aperture.

To ensure constructive interference at the focal point, the optical path length difference (OPD) between rays emerging from different positions of the metalens aperture should not exceed the coherence length of the illumination. Considering the marginal ray (at the lens edge) and the on-axis ray, the OPD at the focus can be approximated as:

$$\varDelta L = \sqrt{f^2 + r} - f = r\left(\frac{1}{\sin\theta} - \frac{1}{\tan\theta}\right) = r\frac{1-\cos\theta}{\sin\theta} = r\tan\left(\frac{\theta}{2}\right) \leq L_c \qquad (S12)$$

where r is the effective radius of the metalens aperture, f is the (wavelength-dependent) focal length, and θ is the focusing half-angle corresponding to the marginal ray.
The coherence length is related to the spectral resolution of the spectrometer. Up to a constant factor that depends on the coherence length definition, we use the commonly adopted estimate

$$L_c = \frac{\lambda^2}{\delta\lambda} \qquad (S13)$$

where $\delta\lambda$ is the spectral resolution of the spectrometer. Using the definition of numerical aperture $NA = n\sin\theta$ (with n =1 in air), into Eq. (S12) becomes:

$$r\tan\left(\frac{\theta}{2}\right) = r\left(\frac{1-\sqrt{1-NA^2}}{NA}\right) \leq \frac{\lambda^2}{\delta\lambda} \qquad (S14)$$

Equivalently, we obtain the constraint of the redius on the allowable spectral resolution:

$$r \leq \frac{\lambda^2}{\delta\lambda\left(\frac{1-\sqrt{1-NA^2}}{NA}\right)} \qquad (S15)$$
